# Further Extension of Lifespan by *Unc-43/CaMKII* and *Egl-8/PLCβ* Mutations in Germline-Deficient *Caenorhabditis elegans*

**DOI:** 10.3390/cells11223527

**Published:** 2022-11-08

**Authors:** Hildegard I. D. Mack, Laura G. Buck, Sonja Skalet, Jennifer Kremer, Hao Li, Elisabeth K. M. Mack

**Affiliations:** 1 Institute for Biomedical Aging Research, Leopold-Franzens-Universität Innsbruck, 6020 Innsbruck, Austria; 2 Department of Hematology, Oncology and Immunology, Philipps-University Marburg, 35043 Marburg, Germany; 3 Department of Biochemistry and Biophysics, University of California, San Francisco, CA 94158, USA

**Keywords:** aging, stress resistance, insulin signaling, germline stem cells, RNA-seq, collagen

## Abstract

Reduction of insulin/insulin-like growth factor 1 (IGF1) signaling (IIS) promotes longevity across species. In the nematode *Caenorhabditis elegans*, ablation of germline stem cells (GSCs) and activity changes of the conserved signaling mediators *unc-43/CaMKII* (calcium/calmodulin-dependent kinase type II) and *egl-8/PLCβ* (phospholipase Cβ) also increase lifespan. Like IIS, these pathways depend on the conserved transcription factor *daf-16/FOXO* for lifespan extension, but how they functionally interact is unknown. Here, we show that altered *unc-43/egl-8* activity further increases the lifespan of long-lived GSC-deficient worms, but not of worms that are long-lived due to a strong reduction-of-function mutation in the insulin/IGF1-like receptor *daf-2*. Additionally, we provide evidence for *unc-43* and, to a lesser extent, *egl-8* modulating the expression of certain collagen genes, which were reported to be dispensable for longevity of these particular *daf-2* mutant worms, but not for other forms of longevity. Together, these results provide new insights into the conditions and potential mechanisms by which CaMKII- and PLCβ-signals modulate *C. elegans* lifespan.

## 1. Introduction

Studies in invertebrate model organisms, such as the roundworm *Caenorhabditis elegans* and the fruit fly *Drosophila melanogaster*, provided key insights into the biology of lifespan regulation that apparently are also applicable to human aging [1]. A prototypic example is provided by reduced insulin/insulin-like growth factor 1 (IGF1) signaling (IIS). IIS was first recognized in *C. elegans* to cause a dramatic, ~two-fold increase in lifespan and, subsequently, was confirmed to promote longevity in mammals [1]. In *C. elegans*, lifespan extension by reduced IIS, e.g., by reduction-of-function mutations in the *daf-2* gene (the common ortholog of mammalian insulin and IGF1 receptors), is dependent on *daf-16* (the ortholog of mammalian FOXO transcription factors) [1]. Of note, genetic variants in *FOXO3* have been repeatedly and robustly associated with lifespan in humans [2].

Beyond *daf-2* mutation, additional mechanisms for lifespan extension have been described in *C. elegans*, including two pathways that can even further extend the extraordinary long lifespan of *daf-2* mutant worms: the absence of germline stem cells (GSCs) [3,4], and a gain-of-function mutation in the calcium/calmodulin-dependent kinase type II (CaMKII), *unc-43* [5]. This further extension of *daf-2* lifespan raises the possibility that the germline- and the *unc-43* signaling pathways are at least in part mechanistically different from the IIS/*daf-2* pathway. Interestingly, both the germline- and the *unc-43* pathway, just as the *daf-2* pathway, depend on *daf-16* for lifespan extension [3,5]. Whether other factors that promote longevity in GSC- and in *daf-2* deficient animals, such as *hlh-30 (TFEB)*, *hsf-1 (HSF1)*, and *skn-1 (NRF2)* [6,7,8,9], are also shared with the *unc-43* pathway has not yet been investigated.

Several lines of evidence indicate that activation and function of the FOXO-transcription factor DAF-16 differ between *GSC(−)* and *daf-2(−) C. elegans*. For example, only *GSC(−)* worms require the conserved adaptor protein KRI-1 and the translation elongation factor TCER-1 for lifespan extension [10,11]. Moreover, whilst DAF-16 acts in the intestine to ensure *GSC(−)* longevity, it acts in the intestine and neurons to ensure *daf-2(−)* longevity [12]. DAF-16/FOXO function is strongly regulated by multiple protein kinases [13], including, but not limited to, the CaMKII-ortholog UNC-43. UNC-43 promotes DAF-16 nuclear localization by direct phosphorylation at a site different from the sites at which DAF-16 is phosphorylated and inhibited by the kinase AKT downstream of IIS [5]. CaMKII, in turn, is activated upon a sudden increase in intracellular calcium ion (Ca^2+^) levels by binding to Ca^2+^-calmodulin complexes [14]. Many additional mediators of Ca^2+^-signaling are conserved across metazoans and present in *C. elegans*, including multiple types of sarcoplasmic/endoplasmic reticulum or plasma membrane channels, G-protein coupled receptors (GPCRs), phospholipase C (PLC) enzymes, and Ca^2+^-pumps [15]. Among these, the G-protein α-subunit *egl-30*, the γ-aminobutyric acid type B (GABA_B_) receptor *gbb-1*, and the PLCβ-ortholog *egl-8*, through their function in neurons, also contribute to intestinal *daf-16* activation and longevity [16,17,18].

Given that both, germline-ablation and *unc-43* gain-of-function mutation, activate DAF-16 and further extend *daf-2(−)* lifespan, we hypothesized that the two pathways are largely overlapping. We tested this hypothesis in the present study and provide evidence for a novel longevity-promoting mechanism triggered by *unc-43(gf)* and also, albeit more weakly, by *unc-43* and *egl-8* loss, which may explain their different effects on various lifespan-extending pathways in *C. elegans*.

## 2. Materials and Methods

### 2.1. C. elegans Strains and Culture

Strains used in this study are listed in Supplementary Table S1. Worms were cultured following standard protocols on NG agar plates seeded with *E. coli* OP50 [19]. Worms carrying the *glp-1(e2144ts)* mutation served as a genetic model for germline ablation [4,9]. To eliminate germ cells, *glp-1(ts)* strains (referred to as *GSC(−)* in Results/Discussion), and corresponding *glp-1(+)* (i.e., *GSC(+)*) control strains were incubated at 25 °C for the first 24 h of postembryonic development and subsequently shifted to 20 °C for the remainder of the experiment. *daf-2(e1370)* worms and corresponding *daf-2(+)* control worms were continuously cultured at 20 °C. For experiments, all strains were routinely cultured on plates containing 20 µM 5-Fluoro-2′-deoxyuridine (FUDR; Sigma-Aldrich/Merck, Munich, Germany) from L4 onward to avoid progeny development and internal hatching in fertile strains carrying the *unc-43(gf)* mutation *(allele n498)*.

### 2.2. Lifespan Analysis

Worms were synchronized by hypochlorite treatment, cultured under the appropriate temperature regimen (cf. above), and scored for survival every other day starting on days 8–10 of adulthood. From the late L4 stage onward, animals were maintained on 6 cm plates at a density of 40 worms/plate and every 10 days, they were transferred to fresh OP50-seeded FUDR-containing NG agar plates to prevent progeny development and desiccation, respectively. Worms were considered dead if they did not respond to gentle touching with a worm pick. Animals that showed a protruding vulva or had ruptured, died from internal progeny hatching (bagging), or escaped from the plate were censored. 

### 2.3. Stress Resistance Assays

Worms were synchronized by hypochlorite treatment, cultured under the appropriate temperature regimen (cf. above), and transferred to assay plates on day 2 of adulthood (20–30 worms per 3 cm plate for heat stress experiments, 50–60 worms per 3 cm plate for oxidative stress experiments). Survival was scored every 1–2 h. Oxidative stress assay plates contained 15.4 mM *tert*-butyl hydroperoxide (TBHP) and were prepared 12 h before starting the experiment.

### 2.4. Growing Worms for RNA-Extraction

To obtain synchronized populations, gravid adults were treated with hypochlorite, and eggs were allowed to hatch in M9 overnight. ~700 L1 larvae per strain were plated on 10 cm NG agar plates seeded with concentrated *E. coli* OP50 (for RNA-seq: 1600 worms/2 plates) and cultured at the required temperatures (cf. above). At the L4-stage, 500 worms per strain were manually transferred to two *E. coli* OP50-seeded 6 cm NG agar plates supplemented with 20 µM FUDR to inhibit germ cell proliferation and progeny production (for RNA-seq: 1500 worms to two 10 cm plates). At day 1 of adulthood, worms were harvested by washing them off their plates with M9 buffer. After additional washing with M9 and RNAse-free water, worms were suspended in 1 mL Trizol, snap-frozen in liquid nitrogen, and stored at −80 °C until RNA extraction. 

### 2.5. RNA-Extraction

RNA was extracted using Trizol and cleaned up with the Monarch Total RNA Miniprep Kit or RNA Cleanup Kit (New England Biolabs, Ipswich, MA, USA) according to the manufacturer’s instructions. Equal amounts of RNA extracted from three biological replicates were pooled for RNA-sequencing to mitigate batch effects.

### 2.6. RNA-Sequencing

RNA quality control, library preparation, and 50-bp paired-end RNA-sequencing on the Illumina HiSeq4000 platform were performed at Eurofins Genomics, Ebersberg, Germany. For each sample, a minimum of 31.6 × 10^6^ reads (maximum 39.1 × 10^6^, median 37.4 × 10^6^ reads) were obtained, resulting in at least 15.7× genome coverage (maximum 19.5×, median 18.7×). A minimum of 94.5% of reads (maximum 96.3%, median 95.7%) could be mapped to the *C. elegans* reference genome (cf. below).

### 2.7. RNA-Seq Data Analysis

The following tools provided by the European Galaxy Server (Freiburg Galaxy Team, Freiburg, Germany), at https://usegalaxy.eu (accessed on 30 May 2020) were used for the initial analysis steps: *FastQC* (v0.72) and *MultiQC* (v1.7) for initial quality control; *Trimmomatic* (v0.36.5; ILLUMINACLIP with default settings, LEADING:20, TRAILING:20, SLIDINGWINDOW:5:20, MINLENGTH:20) for gentle trimming of reads; *STAR* (v2.7.2b) for alignment to the *C. elegans* reference genome (WS276 release); and *featureCounts* (v1.6.4) for read summarization [20,21,22,23]. All subsequent analysis steps were performed using Bioconductor (v3.12; [24]) and custom R scripts (v4.0.4; R core team, Vienna, Austria). Differential expression analysis was performed using *NOIseq* (v2.34.0; [25,26]). Low counts (<5) were filtered by the CPM method, and replicate simulation was performed using TMM- or UQUA-normalization [27], and otherwise default settings of *NOIseq-sim*. Differentially expressed genes (DEGs) were defined as having a probability of differential expression >95%. Only DEGs that were detected in 20 repetitions of replicate simulation and that displayed an expression-fold chance ≥1.5 with each of the two normalization methods in each of the three repetitions of the entire *NOIseq*-analysis were considered further. PCA-analysis of CPM-filtered, TMM/UQUA-normalized RNA-seq count data was performed using the *NOIseq* PCA function. Heatmaps of filtered, normalized, log-transformed count data or of sample-to-sample distances were generated using the R package pheatmap (v1.0.12). Complete *NOIseq*-data is provided in Supplementary Table S14. Supplementary Tables S15 and S16 list *NOIseq* M and probability values for all DEGs, along with gene ontology (GO) term information.

### 2.8. Overlap of DEG-Lists

The statistical significance of overlaps between gene lists was calculated as the hypergeometric probability of detecting at least as many common genes as observed in the two lists using the *phyper* function in R. Representation factors were calculated as the number of overlapping genes divided by the expected number of overlapping genes in the two lists (http://nemates.org/MA/progs/overlap_stats.html; accessed on 31 August 2020). For all calculations, the number of genes in the genome was set to 10,602, i.e., the number of genes in Wormbase WS276 that passed CPM-filtering during *NOIseq*-analysis. DEG-lists from published studies were, if necessary, converted to WBGene-IDs using WormMine (http://intermine.wormbase.org/tools/wormmine/begin.do; accessed on 10 August 2020) and adjusted to genes in WS276 using custom R-scripts and manual curation. 

### 2.9. GO Term Enrichment Analysis and Collagen/Matrisome Classification of DEG-Lists

GO term enrichment analysis was performed using the enrichment tool provided by Wormbase (https://wormbase.org/tools/enrichment/tea/tea.cgi; accessed on 27 May 2021) [28]. The q-value threshold was set to <0.05. GO term categories were retrieved using REViGO ([29]; http://revigo.irb.hr/; accessed on 27 May 2021). For WormCat analysis [30], the respective R package was downloaded and installed as described on www.wormcat.com (accessed on 9 January 2022). Collagen and matrisome classification [31] was performed using the lists provided at http://CeColDB.permalink.cc/ (accessed on 3 January 2022) and http://ce-matrisome-annotator.permalink.cc/ (accessed on 4 January 2022) and custom R scripts.

### 2.10. Dauer Analysis

Synchronized (hypochlorite treatment) L1 larvae were plated at a density of ~50 larvae/6 cm plate (2 plates per strain), incubated at 20 °C or 25 °C, and inspected for the presence of dauers every 12 h. At the 60 h (20 °C) or 48 h (25 °C) timepoint, when wildtype worms reached adulthood, worms were morphologically classified as “dauer” or “non-dauer”. *Daf-2(−)* strains, which all formed 100% dauers when incubated at 25 °C, were allowed to recover from dauer by incubation at 15 °C for 120 h before classification. Experiments in *daf-2(+)* strains were either conducted in parallel with experiments in *daf-2(−)* strains or included *daf-2(−)* control plates to provide a reference for the typical dauer morphology.

### 2.11. qPCR

A total of 1 µg total RNA was reverse transcribed using LunaScript RT SuperMix (New England Biolabs, Ipswich, MA, USA). qPCR-reactions were performed in duplicates or triplicates in a 20 µL reaction volume on an CFX Connect Real-Time PCR Detection System (Bio-Rad Laboratories, Hercules, CA, USA) with iTaq Universal SYBR Green Supermix (Bio-Rad Laboratories). The thermal cycling protocol comprised one activation step at 95 °C for 3 min, followed by 40 cycles of denaturation at 95 °C for 10 s and combined annealing/extension at 60 °C for 30 s. Melting curve analysis was performed from 65 °C to 95 °C with 0.5 °C increments at 5 s per step. Data were analyzed by the ΔΔCt method, and target gene expression levels were normalized to the geometric means of *cdc-42*, *tba-1*, and *Y45F10D.4* expression [32,33]. Primer sequences are listed in Supplementary Table S2.

### 2.12. Statistical Analysis

Statistical analysis was performed using Prism 5 or 9 (GraphPad Software, San Diego, CA, USA). Details on the particular tests used are specified in the figure legends.

## 3. Results

### 3.1. Unc-43(gf) and Unc-43(−) Further Extend Lifespan of Germline-Deficient C. elegans

**Figure 1:**
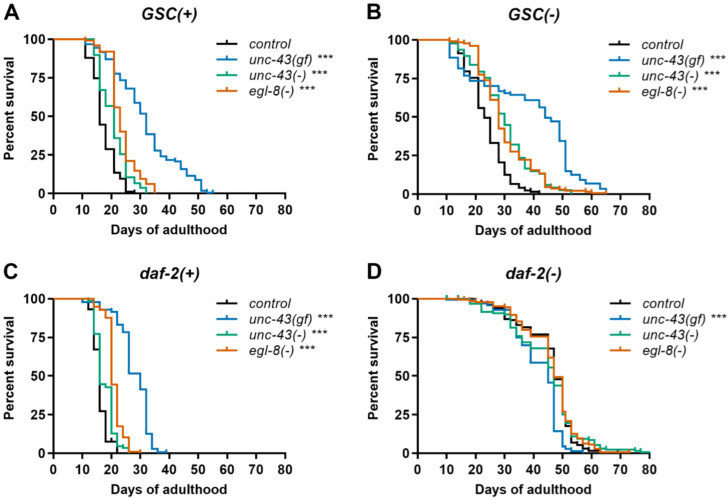
Modulating *CaMKII-* or *PLCβ-*activity extends lifespan in wildtype and *GSC(−)*, but not in *daf-2(−) C. elegans*. Lifespan analysis was performed on (**A**) *GSC(+)*, (**B**) *GSC(−)*, (**C**) *daf-2(+)*, and (**D**) *daf-2(−)* strains carrying mutations in *CaMKII/unc-43* or *PLCβ/egl-8* or no additional mutation (control) as indicated. *Egl-8(−)* refers to allele *md1971*. Data shown are representative of three independent experiments, each comprising ≥ 74 worms per strain. *** indicates *p* < 0.001 (Mantel–Cox test). See Supplementary Table S3 for complete statistical analysis.

To test the hypothesis that germline ablation extends *C. elegans* lifespan at least in part by activating *unc-43/CaMKII*, we generated a set of relevant double mutants and measured their lifespans. Thereby, we took advantage of a widely used genetic model for germline stem cell deficiency, *glp-1(e2144ts)* (hereafter referred to as *GSC(−);* [4]) and combined this allele with the previously studied [5] *unc-43* alleles *n498* and *n498n1186* (referred to as *unc-43(gf)* and *unc-43(−)*, respectively). All experiments were conducted in the presence of FUDR (cf. Materials and Methods), to avoid internal progeny hatching in fertile *unc-43(gf)* worms and to facilitate comparison with similar previous studies that also took advantage of FUDR to prevent progeny development in fertile strains [5,8,17,18,34]. In *GSC(+)*, i.e., otherwise wildtype worms, *unc-43(gf)* robustly extended lifespan (36–87%; Figure 1A, Supplementary Table S3), consistent with a previous report [5]. However, in contrast to this previous report, *unc-43(−)* also consistently produced a small to moderate lifespan increase in otherwise wildtype worms (6–21%; Figure 1A, Supplementary Table S3). Similarly, in *GSC(−)* worms, *unc-43(gf)* substantially extended lifespan (67–92%), while *unc-43(−)* had a more modest effect (2/3 experiments; 22–24%; Figure 1B, Supplementary Table S3). Collectively, these data cannot easily be reconciled with our original hypothesis and instead indicate that *unc-43(gf)* and *unc-43(−)* both trigger longevity-promoting mechanisms. Moreover, these mechanisms apparently are not, or are not maximally, triggered by germline ablation.

### 3.2. Loss of Egl-8 Also Extends Lifespan of Germline-Deficient C. elegans

In parallel with the *unc-43* alleles, we investigated how loss of *egl-8/PLCβ* influences the lifespan of *GSC(−)* worms. As *unc-43(−)*, *egl-8(md1971)*, a putative loss-of-function allele, extended the lifespan of otherwise wildtype worms (13–38%; Figure 1A, Supplementary Table S3), in agreement with published work [16,17,18]. Moreover, *egl-8(md1971)* further extended lifespan in *GSC(−)* worms (30–43%, Figure 1B, Supplementary Table S3). Similar results were obtained with another putative loss-of-function allele, *egl-8(e2917)* (Supplementary Figure S1A,B, Supplementary Table S3). Thus, *egl-8(−)*, similar to *unc-43(−)* and *unc-43(gf)* appears to induce lifespan-extending programs that are not, or not fully, active in *GSC(−)* worms.

### 3.3. Unc-43(gf), Unc-43(−) and Egl-8(−) Do Not Further Extend Lifespan of Daf-2(−) Worms

In addition to our lifespan analyses on *unc-43(gf)*, *unc-43(−)* and *egl-8(−)* in intact and in GSC-deficient animals, we examined the effect of these alleles on the lifespan of worms harboring a strong reduction-of-function mutation in the insulin/IGF-1 like receptor *daf-2* (allele *e1370*) [35]. As mentioned above, in wildtype (*daf-2(+)*) animals, *unc-43(gf)* substantially and *unc-43(−)* more modestly extended lifespan (34–82% and 5–23%; Figure 1C, Supplementary Table S3). In *daf-2(−)* worms, however, unexpectedly in the light of published work by others [5], *unc-43(gf)* did not further extend but rather slightly decrease lifespan (9–13%; Figure 1D, Supplementary Table S3). Moreover, *unc-43(−)* did not significantly decrease *daf-2(−)* lifespan. Consistent with our previous observations (Figure 1A), both *egl-8(−)* alleles extended lifespan in *daf-2(+)* worms (27–39%; Figure 1C, Supplementary Figure S1C, Supplementary Table S3). Finally, *egl-8(−)*, similar to *unc-43(−),* had essentially no effect on *daf-2(−)* lifespan (Figure 1D, Supplementary Figure S1D, Supplementary Table S3). In summary, irrespective of the discrepancies with previously published work [5], our results suggest that *unc-43(gf), unc-43(−)* and *egl-8(−)* all trigger longevity-promoting pathways that are already highly active, or that are dispensable for longevity, in *daf-2(−)* worms.

### 3.4. Unc-43(gf), Unc-43(−) and Egl-8(−) Differentially Affect Stress Resistance of Wildtype, GSC(−) and Daf-2(−) Worms

**Figure 2:**
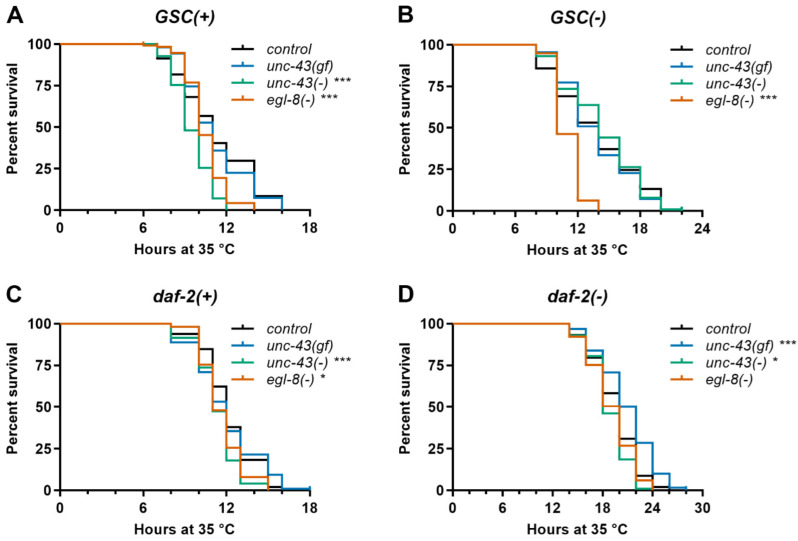
*CaMKII* and *PLCβ* modulate *C. elegans* heat stress resistance in a genetic background-dependent manner. Survival at 35 °C was scored for (**A**) *GSC(+)*, (**B**) *GSC(−)*, (**C**) *daf-2(+)*, and (**D**) *daf-2(−)* strains carrying mutations in *CaMKII/unc-43* or *PLCβ*/*egl-8* or no additional mutation (control) as indicated. *Egl-8(−)* refers to allele *md1971*. Data shown are representative of three independent experiments, each comprising ≥ 75 worms per strain. *** indicates *p* < 0.001, * *p* < 0.05 (Mantel–Cox test). See Supplementary Table S4 for complete statistical analysis.

**Figure 3:**
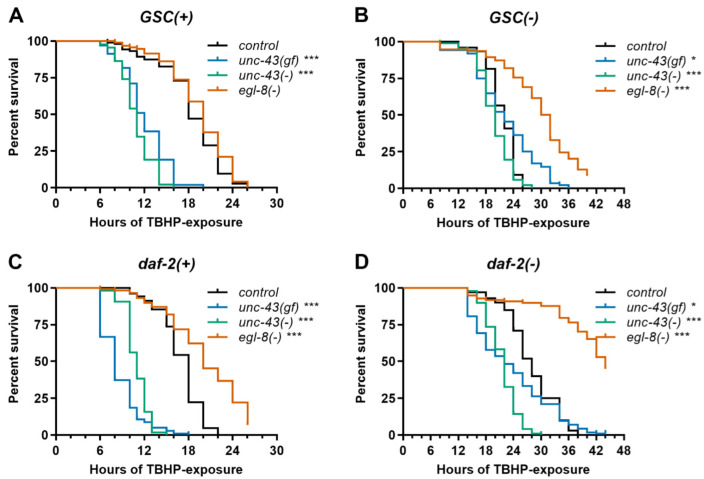
*CaMKII-* and *PLCβ* modulate *C. elegans* oxidative stress resistance in a genetic background-dependent manner. Survival in the presence of TBHP was scored for (**A**) *GSC(+)*, (**B**) *GSC(−)*, (**C**) *daf-2(+)*, and (**D**) *daf-2(−)* strains carrying mutations in *CaMKII/unc-43* or *PLCβ/egl-8* or no additional mutation (control) as indicated. *Egl-8(−)* refers to allele *md1971*. Data shown are representative of four independent experiments, each comprising ≥ 70 worms per strain. *** indicates *p* < 0.001, * *p* < 0.05 (Mantel–Cox test). See Supplementary Table S4 for complete statistical analysis.

Longevity frequently correlates with stress resistance [1]. Therefore, we examined how our gain- and loss-of-function alleles in *unc-43* and *egl-8* affect the resistance of *GSC(−)*, *daf-2(−)* and otherwise wildtype worms to selected forms of physical stress. Specifically, we examined heat and TBHP-induced oxidative stress. Only in *daf-2(−)* background, *unc-43(gf)* slightly increased heat stress resistance, while only in otherwise wildtype worms, *unc-43(−)* conferred heat sensitivity (Figure 2, Supplementary Table S4). On the other hand, upon oxidative stress, *unc-43(gf)* and *unc-43(−)* both consistently caused at least trends towards sensitivity in most or all backgrounds, with only the *GSC(−); unc-43(gf)* strains being clearly not sensitive (Figure 3 and Supplementary Table S4). All *egl-8(−)* strains consistently tended to be or were more resistant than controls to oxidative stress but more sensitive to heat stress, with the exception of *daf-2(−); egl-8(−)* strains, which did not consistently show heat sensitivity (Figure 2 and Figure 3, Supplementary Figures S2 and S3, Supplementary Table S4). In summary, in all backgrounds in which they (further) extended lifespan, *unc-43(gf)*, *unc-43(−)* and *egl-8(−)* did not confer broad stress resistance but rather sensitivity to at least one of the two physical stressors examined.

### 3.5. Unc-43(gf) and Egl-8(−) Globally Shift Gene Expression towards Daf-2(−)

**Figure 4:**
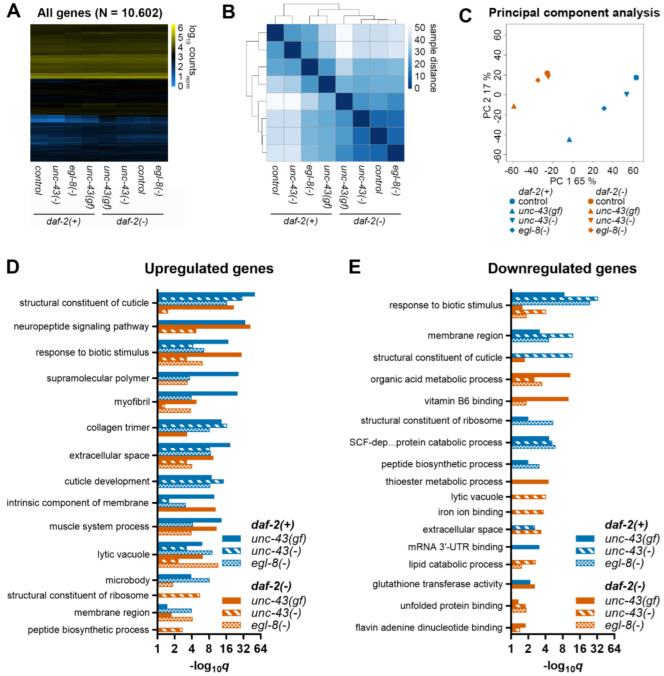
*unc-43(gf)* and *egl-8(−)* globally shift gene expression towards *daf-2(−)*. Gene expression was analyzed by RNA-seq in the strains indicated. (**A**) Heatmap of the filtered, tmm-normalized, log10-transformed RNA-seq count data of all genes evaluated during downstream analysis (cf. Materials and Methods). (**B**) Pairwise distances between the samples (strains) analyzed. (**C**) Principal component analysis of the samples analyzed. (**D**) GO term enrichment analysis among genes upregulated or (**E**) downregulated relative to the wildtype or *daf-2* single mutant control strain in response to the genetic mutations indicated. The graphs show the five most significantly enriched GO terms for each strain, plus their occurrences in all other strains. See Supplementary Table S8 for a complete analysis.

In our hands, *unc-43(−)* and *egl-8(−)* extended *C. elegans* lifespan more modestly, but in the same genetic background-dependent manner as *unc-43(gf)*. To identify novel factors and pathways that may mediate *unc-43′*s and/or *egl-8′*s lifespan-regulatory functions in addition to *daf-16*, we performed an exploratory RNA-sequencing (RNA-seq) study in *daf-2(−)* and otherwise wildtype (*daf-2(+)*) worms with normal or altered *unc-43*/*egl-8* activity. *GSC(−)* worms were omitted from this study since *unc-43*/*egl-8* mutations changed their lifespan in essentially the same way as the lifespan of otherwise wildtype worms. Out of the two *egl-8* loss-of-function alleles, *md1971* was chosen, and hereafter, *egl-8(−)* refers to *md1971* unless otherwise stated. Initial comparison of gene expression between *daf-2(−)* and wildtype worms revealed highly significant overlaps of differentially expressed genes (DEGs) from our study with published work [36,37], thus validating our data (Supplementary Tables S5 and S6). Hierarchical clustering and principal component analysis clearly separated the wildtype and *daf-2(−)* control strains from each other, and strikingly, *egl-8(−)* and especially *unc-43(gf)* single mutant worms appeared closer to *daf-2(−)* in these analyses (Figure 4A–C). In line with this result, genes induced by *unc-43(gf)* and *egl-8(−)* in otherwise wildtype worms significantly overlapped with genes induced by *daf-2(−)* in our and in published studies (Supplementary Table S7). Similarly, *unc-43(gf)*/*egl-8(−)*- and most to all lists of *daf-2(−)* repressed genes significantly overlapped with each other. Interestingly, in otherwise wildtype worms, *unc-43(−)* induced and repressed genes also significantly overlapped with most to all lists of *daf-2(−)* induced and -repressed genes (Supplementary Table S7). In *daf-2(−)* worms, *unc-43(gf)*, *unc-43(−)* and *egl-8(−)* seemed to support some gene expression changes already induced by *daf-2(−)*, while opposing others (Supplementary Table S7). Similar overlap patterns resulted when comparing our lists of *unc-43(gf)*, *unc-43(−)* and *egl-8(−)*-regulated DEGs to genes regulated by *daf-2(−)* in a *daf-16* dependent manner [38,39,40,41] (Supplementary Table S7). Taken together, our RNA-seq results indicate that *unc-43(gf)* and *egl-8(−)* globally shift gene expression towards *daf-2(−)*, and they are consistent with previously reported roles of *unc-43(gf)* and *egl-8(−)* as positive regulators of *daf-16* [5,16,17,18].

### 3.6. Unc-43 and Egl-8 Regulated Genes Are Enriched for Collagen-Related Processes

To functionally categorize *unc-43(gf)*, *unc-43(−)* and *egl-8(−)* DEGs in otherwise wildtype and in *daf-2(−)* worms, we performed gene ontology (GO) term enrichment analysis [28] (Figure 4D,E, Supplementary Table S8). This analysis revealed overrepresentation of GO terms reminiscent of known functions of *unc-43* and *egl-8* in neurons, muscle, and immunity, respectively [16,42]. Moreover, multiple GO terms enriched among DEGs from *unc-43(gf)*, *unc-43(−)* or *egl-8(−)* strains were also enriched among DEGs between *daf-2(−)* and wildtype worms, consistent with the previously observed overlaps between the respective DEG-lists (Supplementary Table S9). Strikingly, the most significantly enriched GO term among upregulated DEGs in all three *daf-2(+)* strains was “structural component of cuticle”, followed by additional GO terms containing many collagen genes (e.g., “collagen trimer”, “cuticle development”, and “molting cycle”), and by multiple GO terms related to pathogen defense. “Structural component of cuticle” and pathogen defense-related GO terms were also enriched among upregulated DEGs of most to all *daf-2(−)* strains. On the other hand, *unc-43(gf)*, *unc-43(−)* and *egl-8(−)* downregulated DEGs were most significantly and commonly enriched for pathogen defense related GO terms in all *daf-2(+)*- and in the *daf-2(−); egl-8(−)* strain, and for organic acid-metabolism-related GO terms in *daf-2(−); unc-43(gf)* and *daf-2(−); unc-43(−)* worms. Enrichment of similar functional categories was detected by a different analysis tool, WormCat [30], including, most prominently among *unc-43(gf)*, *unc-43(−)* and *egl-8(−)*-induced genes, “extracellular material: collagen” (Supplementary Figure S4 and Table S9). Given the conditional requirement of collagens for *C. elegans* longevity [34], we focused on these genes for subsequent analyses.

### 3.7. Unc-43 and Egl-8 Modulate the Expression of Non-Dauer Longevity-Associated Collagen Genes

**Figure 5:**
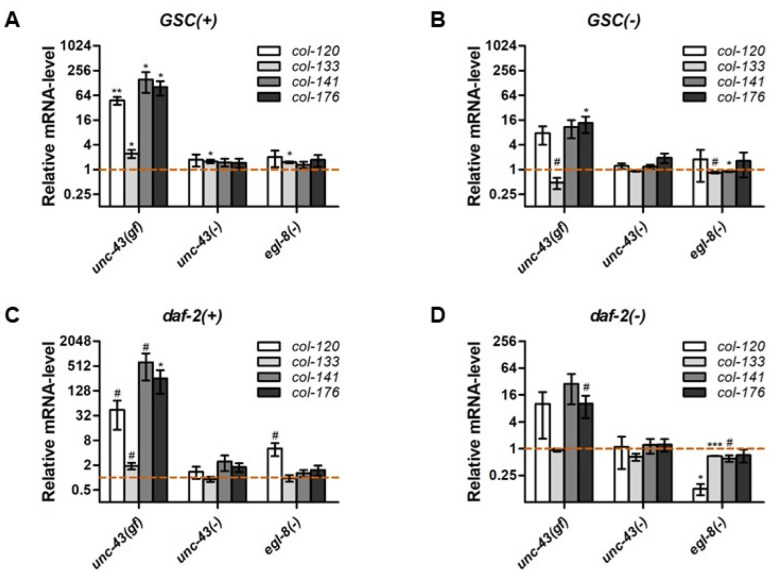
*unc-43* and *egl-8* modulate the expression of non-dauer longevity-associated collagens. mRNA levels relative to the control strain of the same genetic background were determined by qPCR for selected non-dauer longevity-associated collagen genes in (**A**) *GSC(+)*, (**B**) *GSC(−)*, (**C**) *daf-2(+)*, and (**D**) *daf-2(−)* strains carrying the genetic mutations indicated. Bars and error bars indicate mean ± SEM across 3 biological replicates. # indicates *p* < 0.05, * *p* < 0.05, ** *p* < 0.01, *** *p* < 0.001 (unpaired t-tests, FDR 10%). See Supplementary Table S13 for complete statistical analysis.

Certain cuticle-forming collagens have been shown to be required for longevity, specifically under conditions that do not induce processes related to *dauer*, an extremely stress-resistant alternative developmental stage, during adulthood [34]. Expression of these collagens appears to be indirectly controlled by the conserved transcription factor SKN-1/NRF2 [34]. Non-dauer-associated, collagen-dependent lifespan-extending conditions include germline deficiency and certain means of reducing IIS, but not the *daf-2(−)* allele analyzed by us, *daf-2(e1370)*, at least not at the standard culture temperature of 20 °C at which we conducted our study [34]. *Unc-43(gf)*, *unc-43(−)*, and *egl-8(−)* do not sensitize *C. elegans* to dauer entry at 20 °C or 25 °C (Supplementary Figure S5 and Table S9). Therefore, we examined the effect of these alleles on the expression of “SKN-1 upregulated *daf-2(−)* collagens” ([34], subsequently referred to as “non-dauer longevity-associated collagens”). Significant numbers of these collagens were present in our lists of *unc-43(−)*, *egl-8(−)* and especially *unc-43(gf)* upregulated DEGs from *daf-2(+)*, as well as among *unc-43(gf)* upregulated DEGs from *daf-2(−)* worms (Supplementary Tables S10–S12). Up- and downregulated genes from these strains also significantly overlapped with the complete lists of genes up/downregulated by *skn-1* under non-dauer longevity conditions. Yet, only *egl-8(−)* appeared to up- and downregulate significant numbers of genes that are up- or downregulated by *skn-1* under normal conditions (Supplementary Table S11). Classification of collagen-genes [31] induced by *unc-43(gf)*, *unc-43(−)* and *egl-8(−)*, especially in *daf-2(+)* backgrounds, revealed strong overrepresentation of cuticular collagens, as also seen for *skn-1* upregulated (non-dauer longevity-associated) collagens ([34], Supplementary Table S12). When more broadly examining genes of the extracellular matrix, i.e., the matrisome [31], similar patterns were observed as well for *unc-43(gf)*, *unc-43(−)*, *egl-8(−)* and *skn-1(−)* induced DEG-sets, all of which showed particular enrichment of nematode-specific core matrisome genes (Supplementary Table S12). Of note, collagen or core-matrisome genes were not overrepresented in 5/6 published sets of *daf-16*-induced genes (Supplementary Table S12). qPCR analysis of selected non-dauer longevity-associated collagens [34] confirmed the picture suggested by our RNA-seq data: *col-120*, *col-133*, *col-141*, and *col-176* all were strongly upregulated in *unc-43(gf)* relative to otherwise wildtype worms, while induction in *unc-43(−)* and *egl-8(−)* was less consistent and/or weaker (Figure 5A,C, Supplementary Table S13). In *GSC(−)* and *daf-2(−)* worms, *col-120, col-141*, and *col-176* upregulation by *unc-43(gf)* was repeatedly observed, while *unc-43(−)* did not trigger strong alterations (Figure 5B,D, Supplementary Table S13). Additionally, *in daf-2(−)* worms, *egl-8(−)* appeared to at least mildly suppress all four collagen genes examined (Figure 5D, Supplementary Table S13). Relative to wildtype, both *GSC(−)* and *daf-2(−)* worms displayed at least trends towards elevated mRNA levels for most of the collagen genes examined (Supplementary Figure S6). Collectively, these results suggest that *unc-43(gf)*, and to a smaller extent, *unc-43(−)* and *egl-8(−)*, promote the expression of non-dauer longevity-associated collagens. Moreover, the effects of the three alleles appear strongest in *GSC(+)/daf-2(+)*, and weakest, or even lost, in *daf-2(−)* background.

## 4. Discussion

**Figure 6:**
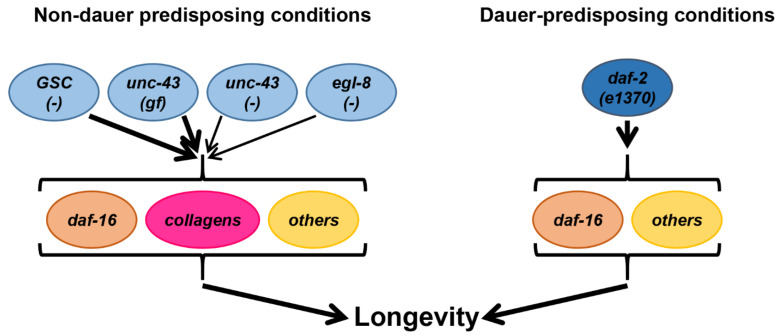
Proposed model for genetic background-dependent lifespan regulation by *unc-43* and *egl-8* mutations. Germline ablation [*GSC(−)*], *unc-43(gf)*, and, more moderately (thin arrows), *unc-43(−)* and *egl-8(−)* promote the expression of at least a subset of specific collagens. These specific collagens have been shown to promote longevity under conditions that do not predispose to dauer, such as *GSC(−)*, and may also contribute to *unc-43(gf)*, *unc-43(−)* and *egl-8(−)*-triggered lifespan extension. Thereby, these specific collagens may act in parallel with other longevity-promoting factors, e.g., *daf-16*. *Daf-16*, but not these specific collagens, is also required for the dauer-predisposing *daf-2(e1370)* mutation to extend lifespan. Note that *daf-16* dependency of lifespan extension has been shown for *GSC(−)*, *unc-43(gf)* and *egl-8(−)*, but not yet for *unc-43(−)*. See the main text for details and references.

In this study, we report lifespan-regulatory functions of the conserved signaling mediators *unc-43* and *egl-8* in long-lived GSC-deficient *C. elegans*. Moreover, our experiments confirm previously published positive roles of *unc-43* hyperactivation and *egl-8* loss for longevity of otherwise wildtype animals and further support the concept that *unc-43* and *egl-8* modulate the activity of the conserved lifespan-regulatory key transcription factor DAF-16 [5,16,17,18]. In addition, gene expression analyses indicate, to at least some extent, the induction of specific non-dauer longevity-associated collagens in response to *unc-43* and *egl-8* mutations. Currently available data do not support a prominent role for *daf-16* in promoting expression of these collagens, raising the interesting possibility that they constitute a second, largely independent mechanism through which *unc-43* and *egl-8* regulate *C. elegans* lifespan (Figure 6).

A key finding of our study consists in the observation that altered *unc-43* and *egl-8* activities further extend the lifespan of wildtype and GSC-deficient but not of *daf-2(−)* (i.e., *daf-2(e1370)* at 20 °C) worms. This result may be explained by at least two, not mutually exclusive, models. First, *unc-43(gf)*, *unc-43(−)* and *egl-8(−)* all may trigger processes that are already maximally induced by *daf-2(−)*, but not by *GSC(−)* and wildtype worms. This model is supported by the fact that all three mutations induce similar gene expression changes than *daf-2(−)*. Alternatively, processes triggered by *unc-43(gf)*, *unc-43(−)* and *egl-8(−)* may not be critical to *daf-2(e1370)* longevity. Consistent with this possibility, *unc-43(gf)* and, to a lesser extent, *unc-43(−)* and *egl-8(−)*, induce the expression of certain collagen genes, which are required for longevity under conditions that do not induce dauer-like traits in adults. These conditions include germline deficiency but not the *daf-2(e1370)* allele under our culture conditions of 20 °C [34]. Thus, it is tempting to speculate that these collagens constitute a second mechanism besides the regulation of *daf-16*, by which in particular *unc-43(gf)*, but also *unc-43(−)* and *egl-8(−)* modulate lifespan. Of note, overexpression of a single key collagen, such as *col-120*, also examined by us by qPCR, is sufficient for lifespan extension [34]. Conversely, in *daf-2(e1370),* mild induction of collagen expression, which is still observed, at least in response to *unc-43(gf)*, may not be functionally relevant. Clearly, it will be extremely interesting to directly examine the role of non-dauer longevity-associated collagens in *unc-43(gf)*, *unc-43(−)* and *egl-8(−)* dependent *C. elegans* lifespan regulation and, eventually, to define the mechanisms that link *unc-43/egl-8* to expression of these collagens. Although we have not yet experimentally confirmed or excluded the possibility that the known mediator of *unc-43(gf)/egl-8(−)* longevity, *daf-16*, regulates non-dauer longevity-associated collagen expression in the specific context of *unc-43/egl-8* mutation, even though it does not do so in other contexts, an alternative candidate is emerging from the literature: *skn-1* [34]. In support of this hypothesis, we already showed that *unc-43(gf), unc-43(−) and egl-8(−)* modulate the expression of similar gene sets, including similar collagens, than *skn-1*. 

Yet, our study and a previous report [5] on the longevity effects of *unc-43(−)* and *unc-43(gf)* differ from each other in several details despite the use of the same alleles. In the case of *unc-43(−)*, these discrepancies may be explained by different experimental conditions, i.e., different temperatures, and by the use of FUDR, both of which have been shown to affect lifespan in certain genetic backgrounds [43,44]. Specifically, we conducted the respective assays at 20 °C rather than 25 °C [5] and analyzed the *unc-43(−)* and *unc-43(gf)* alleles within the same experiments, which required the addition of FUDR not just to *unc-43(gf)* and corresponding control [5], but also to *unc-43(−)* strains. Importantly, FUDR does not affect wildtype lifespan nor *daf-2(−)’s* and *GSC(−)*’s basic ability to promote longevity [8,34,43].

Beyond different assay temperatures, our choice of *daf-2(e1370)*, rather than *daf-2* RNAi [5] to inhibit *daf-2* activity may account for *unc-43(gf)* in our hands not extending the lifespan of worms with reduced IIS. Differential interactions of “strong” (e.g., *e1370*) and “weak” (e.g., RNAi) *daf-2* inhibition with other lifespan-regulatory mechanisms are not unprecedented in the literature, e.g., with somatic gonad signaling and collagen expression [3,34,45]. Moreover, in contrast to genetic mutations, *daf-2* RNAi does not efficiently reduce *daf-2* activity in neurons [12,46,47]. Yet, activity of the common *daf-2(−)*- and *unc-43(gf)* lifespan-extending factors *daf-16* in neurons accounts for a fraction of *daf-2(−)* longevity [12]. Thus, *unc-43(gf)* may trigger *daf-16* regulated processes in neurons that are already active in *daf-2(e1370)*, but not in *daf-2* RNAi worms, which may explain why it further extends *daf-2* RNAi- but not *daf-2(e1370)* lifespan. 

The observation that both, *unc-43(−)* and *unc-43(gf)* extend lifespan, at least in certain genetic backgrounds under certain experimental conditions, appears paradoxical at the first glance. On the other hand, the moderate to strong longevity increase of *unc-43(−)* and *unc-43(gf)* worms can be expected, based on the nature of the—relatively moderate to relatively large—changes in gene expression triggered by these two mutations. Indeed, as discussed above, *unc-43(−)* and *unc-43(gf)* regulated gene sets are similar to each other and to *daf-2*- and *daf-16-*regulated gene sets. In addition, both mutations, although to different extents, induce non-dauer longevity-associated collagens. How *unc-43(−)* and *unc-43(gf)* affect collagen expression and/or its indirect regulator *skn-1* is currently unclear [34]. Previous work [5] suggested UNC-43 as a direct activator of DAF-16, implying that the induction of some *daf-16* targets upon *unc-43(−)* loss, as observed in our RNA-seq study, represents additional, indirect effects of *unc-43* on *daf-16*. Moreover, UNC-43′s direct contribution to DAF-16 activity appears to be dispensable for the maintenance of a normal/normally long lifespan in all genetic backgrounds tested, as *unc-43(−)*, *GSC(−); unc-43(−)* and *daf-2(−); unc-43(−)* worms are long-lived, in contrast to *daf-16(-)*, *GSC(−); daf-16(-)* and *daf-2(−); daf-16(-)* worms, which are short- to normal-lived [3,48]. As proposed previously for reduced IIS [5], GSC loss and other mechanisms induced by *unc-43* loss may “overpower” (direct) DAF-16 regulation by UNC-43.

In agreement with published studies [16,17,18], we found that loss of *egl-8* further extends wildtype lifespan. However, in contrast to previous work [16], we did not observe decreased oxidative stress resistance upon *egl-8* loss. Again, this difference may be explained by different experimental conditions, such as different *egl-8(−)* alleles (*md1971* vs. *n488*), different assay temperatures (20 °C vs. 25 °C) and treatment with different chemicals to impose oxidative stress (TBHP vs. arsenite). Indeed, TBHP and arsenite have already been shown to trigger different transcriptional responses [49]. In a current model, *egl-8* modulates lifespan cell non-autonomously through its function in neurons: *egl-8* loss reduces neuronal secretion of insulin-like peptides, which in turn increases *daf-16* activity in the intestine; in addition, evidence suggests that this pathway is further mediated by the EGL-8-generated second messenger diacylglycerol (DAG) and the DAG-dependent kinase *dkf-2* [16,17,18,50]. This model predicts that worms with compromised insulin/IGF1-like receptor function in the intestine will not display increased longevity upon an *egl-8(−)* mutation, while *GSC(−)* worms may display further intestinal *daf-16* activation and lifespan extension. Results from our lifespan analyses are in line with this prediction. Conceptually, *egl-8* loss also decreases the levels of another second messenger, inositol-1, 4, 5-trisphosphate and subsequently, intracellular Ca^2+^ levels, and CaMKII/UNC-43 activity [15]. Yet, additional studies are necessary to determine how *egl-8(−)* and *unc-43(−)* interact to modulate *daf-16* activity and *C. elegans* lifespan.

In summary, our study identified new genetic backgrounds in which *unc-43* and *egl-8* can/cannot modulate lifespan in *C. elegans* and suggests collagen expression as a second lifespan-regulatory mechanism functioning downstream of *unc-43* and *egl-8*, in addition to the known regulator *daf-16*.

## Data Availability

All relevant data are within the manuscript and its supplementary files. The RNA-sequencing data set generated during the present study has been deposited in the ArrayExpress database at EMBL-EBI (www.ebi.ac.uk/arrayexpress) under accession number E-MTAB-11045.

## Supplementary Materials

The following supporting information can be downloaded at: https://www.mdpi.com/article/10.3390/cells11223527/s1. References [51,52,53,54] are cited within the Supplementary Materials. File S1, containing supplementary table legends and Figure S1: Modulating *PLCβ*-activity extends lifespan in wildtype and *GSC(−)* but not in *daf-2(−) C. elegans*; Figure S2: *PLCβ* modulates *C. elegans* heat stress resistance in a genetic background dependent manner; Figure S3: *PLCβ* modulates *C. elegans* oxidative stress resistance in a genetic background dependent manner; Figure S4: *unc-43(gf), unc-43(−)* and *egl-8(−)* promote the expression of collagens; Figure S5: *unc-43(gf)*, *unc-43(−)* and *egl-8(−)* do not sensitize *daf-2(+)* worms to dauer entry, but *unc-43(gf)* enhances dauer-predisposition of *daf-2(e1370);* Figure S6: Effect of *daf-2*- and GSC-loss on the expression of non-dauer longevity-associated collagens. File S2, containing Table S1: List of strains used in this study; Table S2: List of qPCR primers used in this study; Table S3: Statistical analysis of lifespan data; Table S4: Statistical analysis of stress resistance data; Table S5: Differentially expressed genes detected in this study; Table S6: Overlap of genes regulated by *daf-2(−)* in this study and in published work; Table S7: Overlap of genes regulated by *unc-43/egl-8* and *daf-2/daf-16* in this study and in published work; Table S8: GO term enrichment among *unc-43(gf)*, *unc-43(−)* and *egl-8(−)*-dependent DEGs in *daf-2(+)* and *daf-2(−)* background; Table S9: Statistical analysis of dauer formation data; Table S10: Expression of *skn-1* regulated collagen genes in strains analyzed by RNA-seq; Table S11: Overlap of genes regulated by *unc-43/egl-8* and *skn-1*; Table S12: Classification of collagen- and matrisome genes regulated by *unc-43/egl-8* and comparison to *skn-1*; Table S13: Statistical analysis of qPCR data. File S3, containing Table S14: Complete *NOIseq* analysis of RNA-seq data. File S4, containing Table S15: GO term information and *NOIseq*-data on differentially expressed genes. File S5, containing Table S16: WormCat category information and *NOIseq*-data on differentially expressed genes.
