# Supplementary material for: Further Extension of Lifespan by Unc-43/CaMKII and Egl-8/PLCβ Mutations in Germline-Deficient Caenorhabditis elegans
Source: Cells. 2022 Nov 8;11(22):3527. doi: 10.3390/cells11223527 (PMC9688844; doi:10.3390/cells11223527)
Supplement: Supplementary file 1 [file cells-11-03527-s001.zip › Mack et al_File S1_Legends+Figures S1-S6.pdf]

Supplementary File S1

Further extension of lifespan by *unc-43/CaMKII* and *egl-8/PLCβ* mutations in germline-deficient *Caenorhabditis elegans*

Hildegard I. D. Mack, Laura G. Buck, Sonja Skalet, Jennifer Kremer, Hao Li, Elisabeth K. M. Mack

This supplementary file contains

- Supplementary table legends (Supplementary Tables S1-S13 provided in Supplementary File S2, Supple-mentary Tables S14-S16 provided as separate files)
- Supplementary Figures S1-S6, including figure legends

Supplementary Table legends

Table S1. List of strains used in this study.

Table S2. List of qPCR primers used in this study.

Table S3. Statistical analysis of lifespan data. Accompanies Figure 1 and Supplementary Figure S1. The tables list mean and median survival times in days, standard deviations and standard errors of the mean, the number of worms scored, percent lifespan changes, and p-values from Kaplan-Maier survival analysis relative to same background-control or wildtype, for each biological replicate of the respective experiment. p-values < 0.05 were considered statistically significant.

Table S4. Statistical analysis of stress resistance data. Accompanies Figures 2/3 and Supplementary Figures S2/S3. The tables list mean and median survival times in days, standard deviations and standard errors of the mean, the number of worms scored, percent lifespan changes, and p-values from Kaplan-Maier survival analysis relative to same background-control or wildtype, for each biological replicate of the respective experiment. p-values < 0.05 were considered statistically significant.

Table S5. Differentially expressed genes detected in this study. See Materials and Methods for details on RNA-seq data analysis.

Table S6. Overlap of genes regulated by *daf-2(-)* in this study and in published work. For each overlap, the table list the number of overlapping genes (line 1), the enrichment factor (line 2) and hypergeometric probability of achieving an overlap of at least this size (line 3, italicized). p-values < 0.05 were considered statistically significant. N_published: number of genes as published in the respective study. N_WS276: number of genes in published studies, adjusted to Wormbase version WS276. N: number of genes in published studies, adjusted to WS276, that are also detected in our *NOIseq*-analysis. i.e. all genes that passed low count-filtering.

Table S7. Overlap of genes regulated by *unc-43/egl-8* and *daf-2/daf-16* in this study and in published work. See legend to Supplementary Table S6.

Table S8. GO term-enrichment among *unc-43(gf)*, *unc-43(-)* and *egl-8(-)*-dependent DEGs in *daf-2(+)* and *daf-2(-)* background. Accompanies Figures 4D, E and Supplementary Figure S4. *unc-43(gf)*, *und-43(-)* and *egl-8(-)* induced and -repressed DEGs determined by RNA-seq in the genetic backgrounds indicated were subjected to GO term enrichment analysis by the method indicated in the respective sub-table. q-values/corrected p-values < 0.05 were considered statistically significant. GO terms enriched among DEGs from *daf-2(-)* vs *daf-2(+)* worms were not plotted in the figures to enhance clarity.

Table S9. Statistical analysis of dauer formation data. Accompanies Supplementary Figure S5. The tables list the number of worms examined and classified as dauers/non-dauers for each biological replicate of the respective experiment. Statistical significance was determined by one-way repeated measures ANOVA with Holm-Šídák multiple comparison tests. p-values < 0.05 were considered statistically significant.

Table S10. Expression of *skn-1* regulated collagen genes in strains analyzed by RNA-seq. Non-dauer longevity-associated collagens were determined by Ewald et al., 2015. For each collagen gene, the table list TMM- and UQUA-normalized counts in each strain, as well as the $\log_2$ fold-change ($\log_2$ FC) and probability of differential expression relative to the same background-control strain as determined by replicate-simulation in *NOIseq*. Genes were considered as differentially expressed if probability of differential expression > 0.95 and $|\log_2 \text{FC}| > 0.58$.

Table S11. Overlap of genes regulated by *unc-43/egl-8* and *skn-1*. See legend to Supplementary Table S6.

Table S12. Classification of collagen- and matrisome genes regulated by *unc-43/egl-8* and comparison to *skn-1*. See legend to Supplementary Table S6.

Table S13. Statistical analysis of qPCR data. Accompanies Figure 5 and Supplementary Figure S6. The tables list number of technical replicates, normalized expression of the *col*-genes indicated relative to wildtype or same genetic background-control (NRE), standard deviations and p-values from one-sample t-tests vs a hypothetical mean of 0 for each biological replicate of the respective experiment. Means across all biological replicates were compared by unpaired t-tests (each mutant vs same background-control strain) and p-values were corrected for multiple comparisons by the two-stage linear step-up procedure of Benjamini, Krieger and Yekutieli, setting the FDR to 10 %. p/q-values < 0.05 were considered statistically significant. Normalized relative expression values compared to the same background control strain, or from comparing *GSC(-)/daf-2(-)* to wildtype, were plotted on a linear scale in the figures.

Table S14. Complete *NOIseq* analysis of RNA-seq data. The table lists expression values (mean), $\log_2$ fold-change (M) and absolute expression difference between the two conditions compared (D), a summary statistic for M and D (ranking), and probability of differential expression (prob) for each of the 10,602 genes that passed low-count filtering (CPM < 5), as determined by one round of replicate simulation in *NOIseq-sim*, using either TMM- or UQUA-normalization. TMM-/UQUA-normalized counts for these genes, as well as raw counts and additional information on all detected features, are also provided. md1971: *egl8(-)*; n1186: *unc-43(-)* (allele *n498n1186*); n498: *unc-43(gf).*

Table S15. GO term information and *NOIseq*-data on differentially expressed genes. For all DEGs (cf. Supplementary Table S5), the table lists GO term associations as determined by Wormbase GO term enrichment analysis, along with selected *NOIseq*-values (M and prob, TMM- and UQUA-normalization). See legend for Table S14 and Materials and Methods for additional information.

Table S16. WormCat category information and *NOIseq*-data on differentially expressed genes. For all DEGs (cf. Supplementary Table S5), the table lists category associations as determined by WormCat analysis, along with selected *NOIseq*-values (M and prob, TMM- and UQUA-normalization). See legend for Supplementary Table S14 and Materials and Methods for additional information.

Figure S1

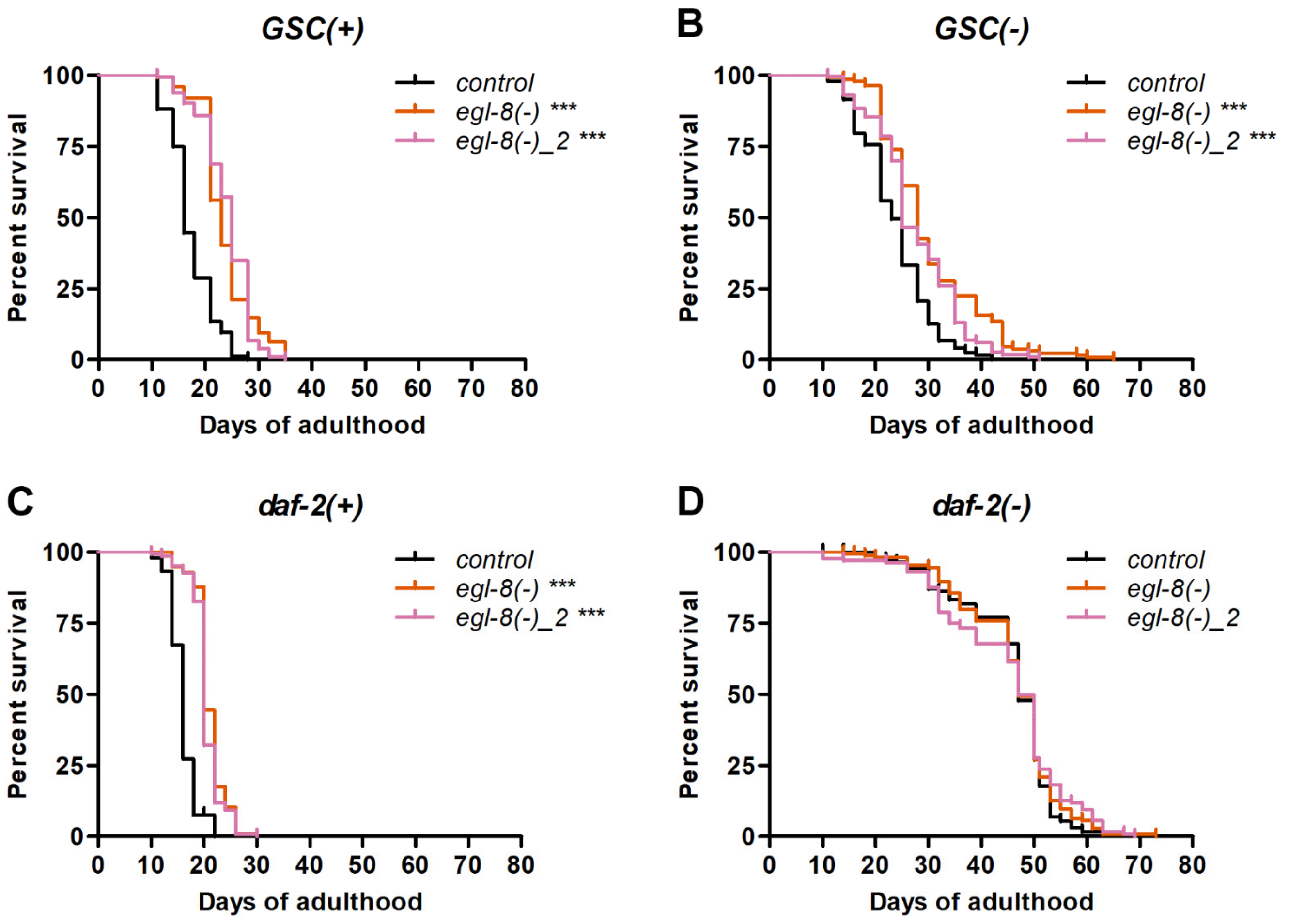

Figure S1. Modulating *PLCβ*-activity extends lifespan in wildtype and *GSC(-)*, but not in *daf-2(-) C. elegans*. Lifespan analysis was performed on (A) *GSC(+)*, (B) *GSC(-)*, (C) *daf-2(+)*, and (D) *daf-2(-)* strains carrying mutations in *PLCβ/egl-8* or no additional mutation (control) as indicated. ***E****gl-8(-)* refers to allele *md1971*, *egl-8(-)_2* to allele *e2917*. Lifespan curves for control and *egl-8(-)* strains (cf. the respective panels of Figure 1) were also plotted here to facilitate comparison. Data shown are representative for 3 independent experiments, each comprising ≥ 74 worms per strain. *** indicates $p < 0.001$ (Mantel-Cox test). See Supplementary Table S3 for complete statistical analysis.

Figure S2

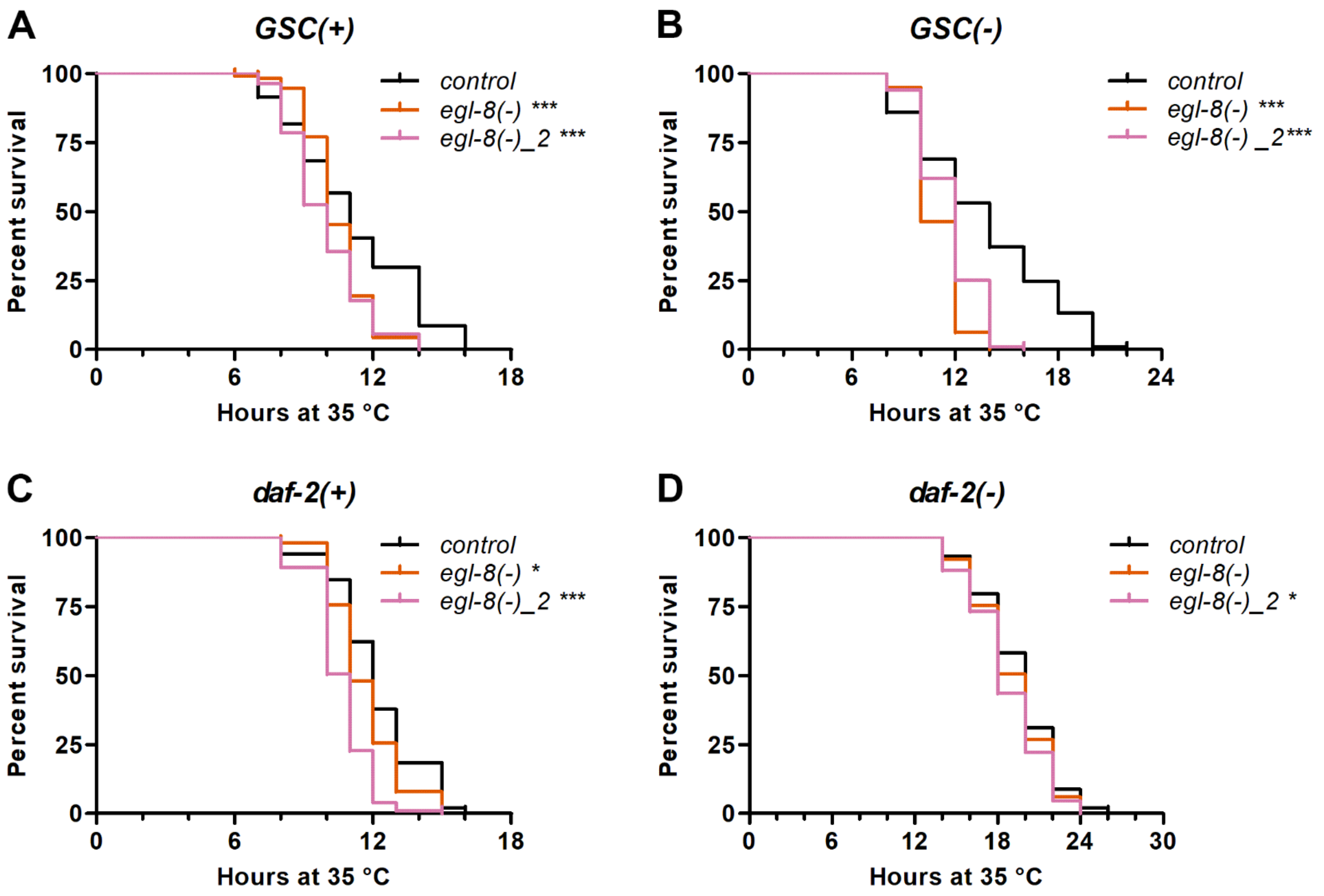

Figure S2. *PLCβ* modulates *C. elegans* heat stress resistance in a genetic background dependent manner. Survival at 35 °C was scored for (**A**) *GSC(+)*, (**B**) *GSC(-)*, (**C**) *daf-2(+)*, and (**D**) *daf-2(-)* strains carrying mutations in *PLCβ*/*egl-8* or no additional mutation (control) as indicated. *Egl-8(-)* refers to allele *md1971*, *egl-8(-)_2* to allele *e2917*. Survival curves for control and *egl-8(-)* strains (cf. the respective panels of Figure 2) were also plotted here to facilitate comparison. Data shown are representative for 2 independent experiments, each comprising ≥ 75 worms per strain. *** indicates $p < 0.001$, * $p < 0.05$ (Mantel-Cox test). See Supplementary Table S4 for complete statistical analysis.

Figure S3

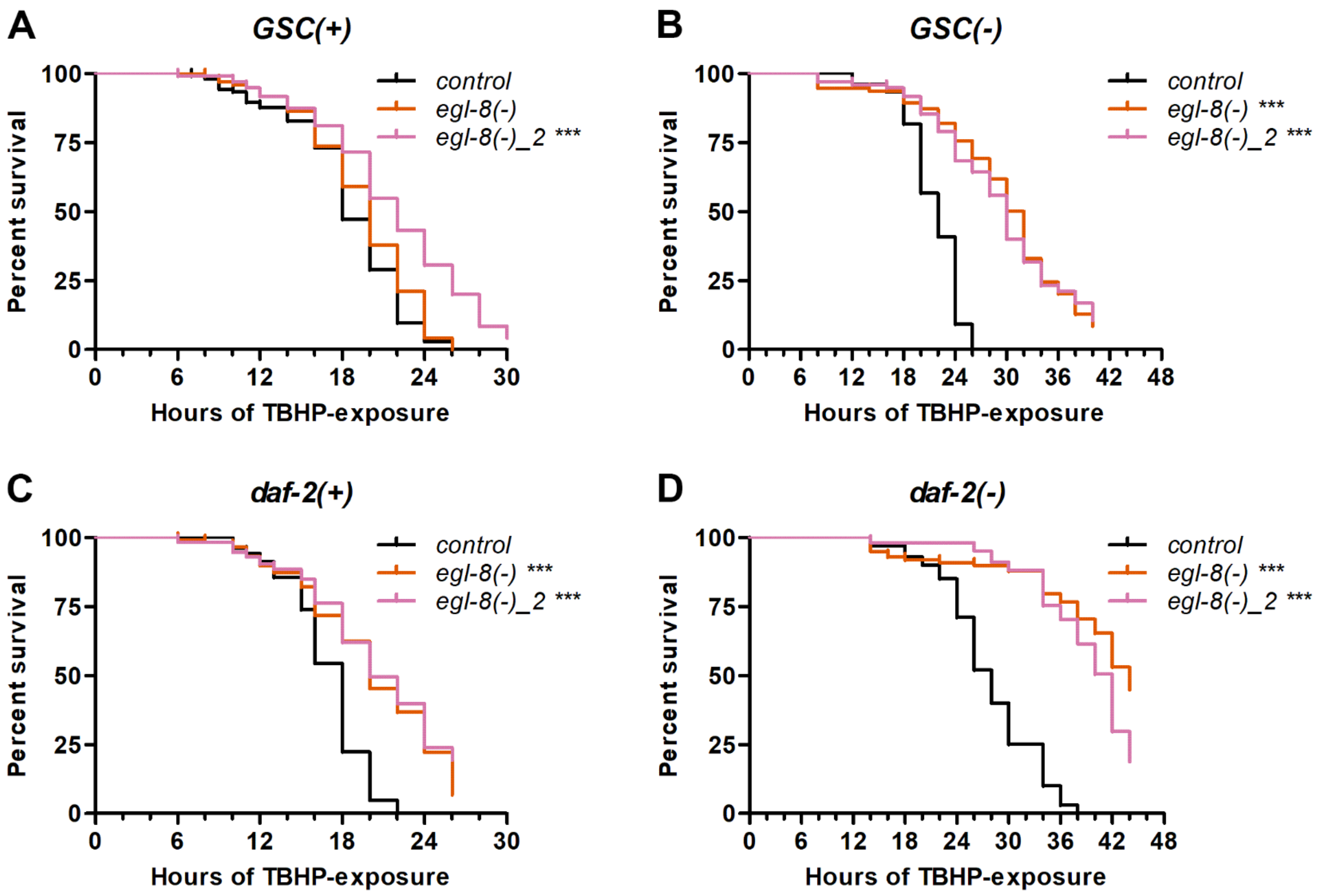

Figure S3. *PLCβ* modulates *C. elegans* oxidative stress resistance in a genetic background dependent manner. Survival in the presence of TBHP was scored for (**A**) *GSC(+)*, (**B**) *GSC(-)*, (**C**) *daf-2(+)*, and (**D**) *daf-2(-)* strains carrying mutations in *PLCβ/egl-8* or no additional mutation (control) as indicated. *Egl-8(-)* refers to allele *md1971*, *egl-8(-)_2* to allele *e2917*. Survival curves for control and *egl-8(-)* strains (cf. the respective panels of Figure 2) were also plotted here to facilitate comparison. Data shown are representative for 2 independent experiments, each comprising ≥ 69 worms per strain. *** indicates $p < 0.001$, * $p < 0.05$ (Mantel-Cox test). See Supplementary Table S4 for complete statistical analysis.

Figure S4

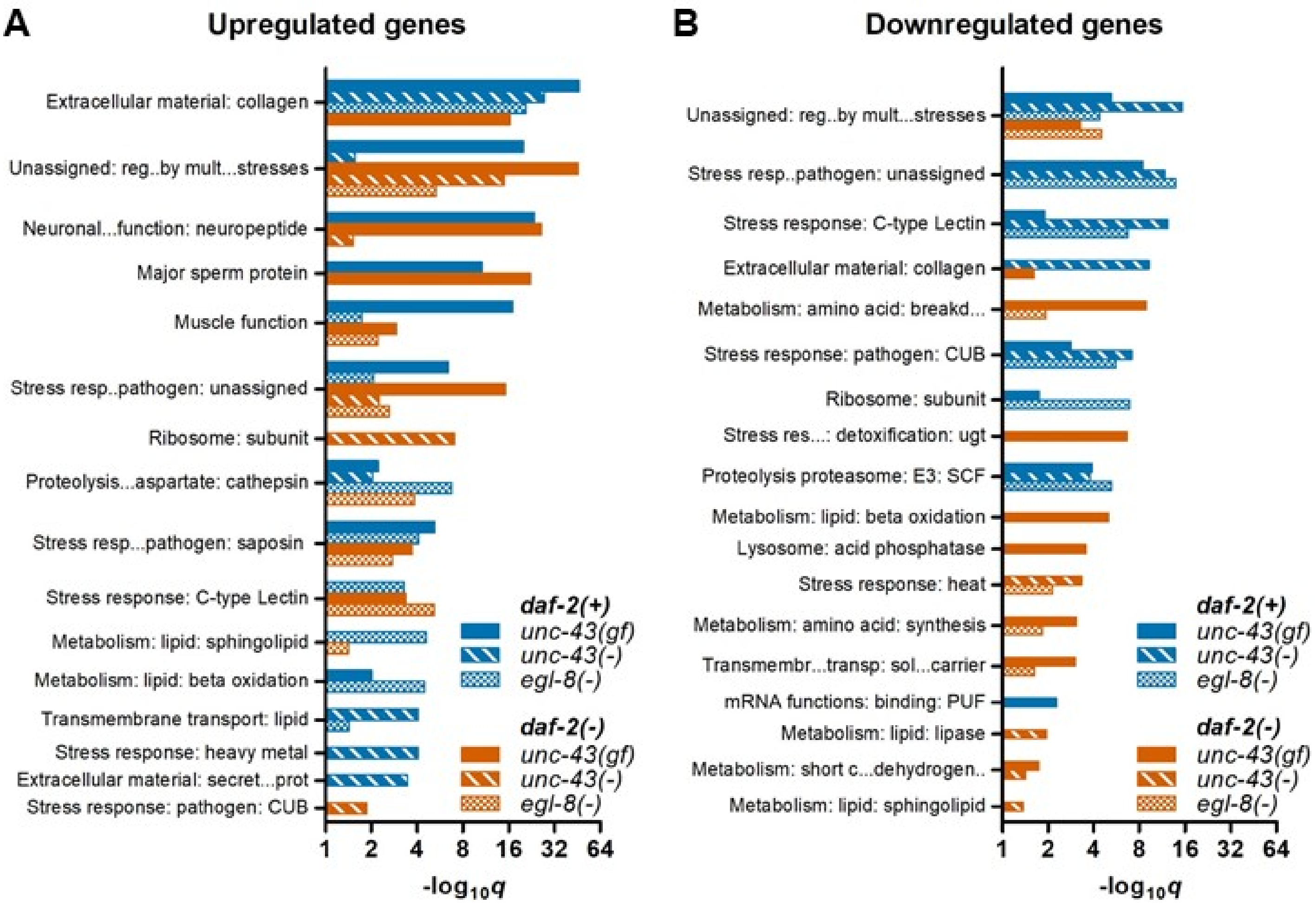

Figure S4. *unc-43(gf), unc-43(-)* and *egl-8(-)* promote the expression of collagens. Functional enrichment of GO terms was analyzed by WormCat among genes (**A**) upregulated or (**B**) downregulated relative to the wildtype or *daf-2* single mutant control strain in response to the genetic mutations indicated. The graphs show the five most significantly enriched GO terms for each strain, plus their occurrences in all other strains. See Supplementary Table S8 for complete statistical analysis.

Figure S5

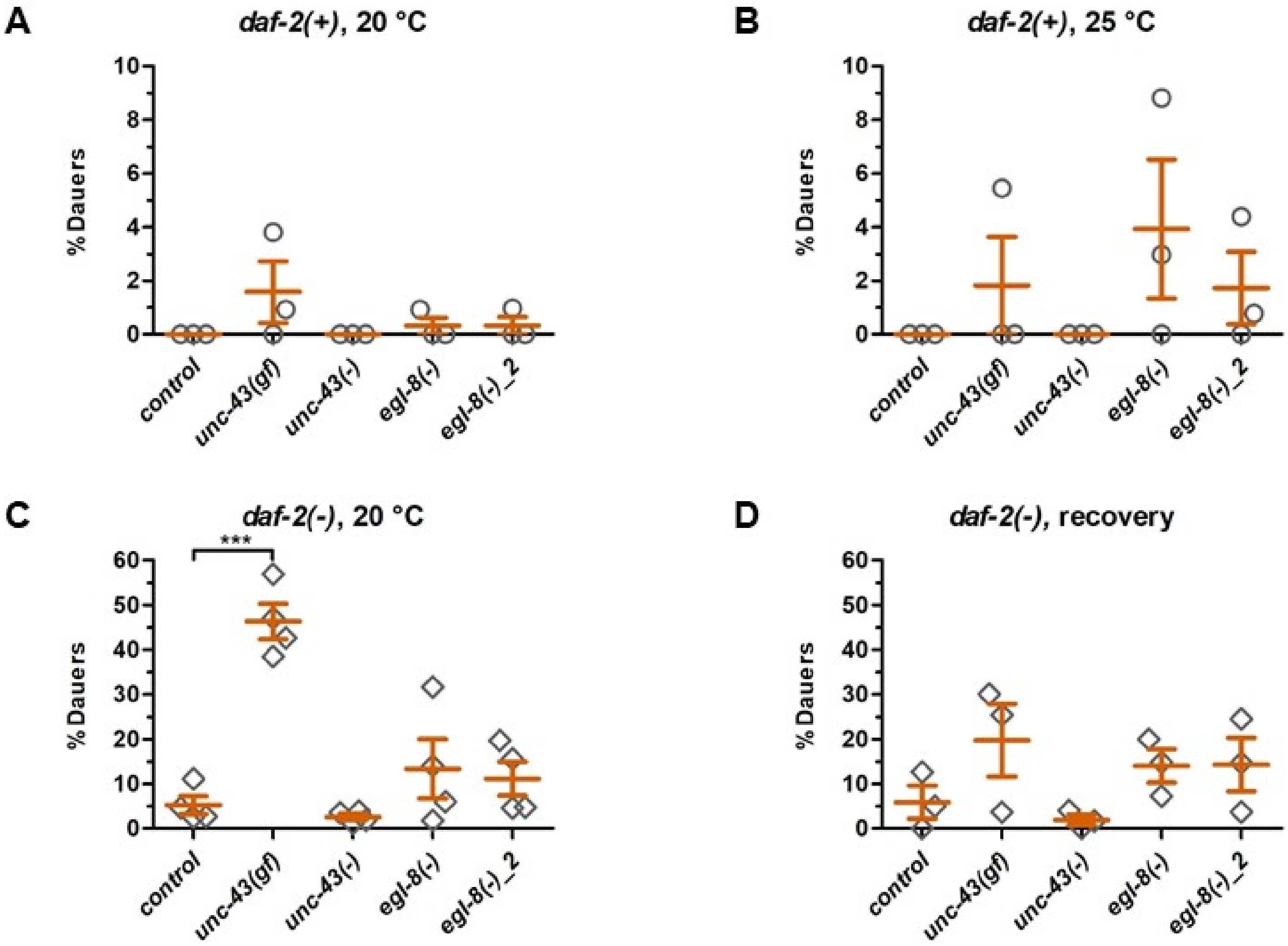

Figure S5. *unc-43(gf)*, *unc-43(-)* and *egl-8(-)* do not sensitize *daf-2(+)* worms to dauer entry but *unc-43(gf)* enhances dauer-predisposition of *daf-2(e1370).* Dauer formation of worms carrying *unc-43*, *egl-8* or no additional mutation (control) as indicated was monitored in various genetic backgrounds under different culture conditions: (**A**) *daf-2(+)* worms at 20 °C; (**B**) *daf-2(+)* worms at 25 °C; (**C**) *daf-2(-)* worms at 20 °C; and (**D**) recovery of *daf-2(-)* strains from dauer induced by incubation at 25 °C. Data indicate mean ± SEM from 3-4 biological replicates. *** indicates $p < 0.001$ (one-way repeated measures ANOVA with Holm-Šídák multiple comparisons test). *egl-8(-)*: allele *md1971*. *egl-8(-)_2*: allele *e2917*. See Supplementary Table S9 for complete statistical analysis.

Figure S6

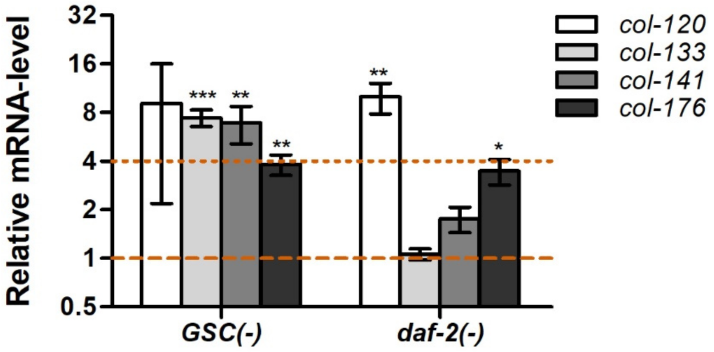

Figure S6. Effect of *daf-2*- and GSC-loss on the expression of non-dauer longevity-associated collagens. mRNA levels relative to the corresponding *daf-2(+)* or *GSC(+)* strain were determined by qPCR for selected non-dauer longevity-associated collagen genes (Ewald et al., 2015). Bars and error bars indicate mean ± SEM across 3 biological replicates. * indicates $q < 0.05$, ** $q < 0.01$, *** $q < 0.001$ (unpaired t-tests with Holm-Šídák multiple comparisons test). Note that other authors (Steinbaugh et al., 2015) have proposed a fold-change of ~4- (dotted line) for detecting genes differentially expressed between *GSC(+)* and *GSC(-)* worms, as they differ in the presence of a germline. See Supplementary Table S13 for complete statistical analysis.
